# Cellulose Acetates in Hydrothermal Carbonization: A Green Pathway to Valorize Residual Bioplastics

**DOI:** 10.1002/cssc.202401163

**Published:** 2024-10-18

**Authors:** Giulia Ischia, Filippo Marchelli, Nicola Bazzanella, Riccardo Ceccato, Marco Calvi, Graziano Guella, Claudio Gioia, Luca Fiori

**Affiliations:** ^1^ Department of Civil Environmental and Mechanical Engineering University of Trento Via Mesiano 77 38123 Trento Italy; ^2^ Department of Physics University of Trento Via Sommarive 14 38123 Trento Italy; ^3^ Department of Industrial Engineering University of Trento Via Sommarive 9 38123 Trento Italy; ^4^ Certottica S.c.r.l. Italian Institute of Certification of Optical Products Villanova Industrial Area 32013 Longarone Italy; ^5^ Center Agriculture Food Environment (C3A) University of Trento Via Edmund Mach 1 38010 San Michele all'Adige Italy

**Keywords:** Acetyl triethyl citrate, Carbon microspheres, Hydrochar, Hydrolysis, Kinetics

## Abstract

Bioplastics possess the potential to foster a sustainable circular plastic economy, but their end‐of‐life is still challenging. To sustainably overcome this problem, this work proposes the hydrothermal carbonization (HTC) of residual bioplastics as an alternative green path. The focus is on cellulose acetate – a bioplastic used for eyewear, cigarette filters and other applications – showing the proof of concept and the chemistry behind the conversion, including a reaction kinetics model. HTC of pure and commercial cellulose acetates was assessed under various operating conditions (180–250 °C and 0–6 h), with analyses on the solid and liquid products. Results show the peculiar behavior of these substrates under HTC. At 190–210 °C, the materials almost completely dissolve into the liquid phase, forming 5–hydroxymethylfurfural and organic acids. Above 220 °C, intermediates repolymerize into carbon‐rich microspheres (secondary char), achieving solid yields up to 23 %, while itaconic and citric acid form. A comparison with pure substrates and additives demonstrates that the amounts of acetyl groups and derivatives of the plasticizers are crucial in catalyzing HTC reactions, creating a unique environment capable of leading to a total rearrangement of cellulose acetates. HTC can thus represent a cornerstone in establishing a biorefinery for residual cellulose acetate.

## Graphical abstract

Pure and commercial cellulose acetates are subjected to hydrothermal carbonization as a possible end‐of‐life strategy. The plasticizers cause the material to completely dissolve in the liquid phase at 190 °C. Valuable carbonaceous solids and platform chemicals were observed in the products, proving the possibility of applying this approach in a biorefinery scenario.

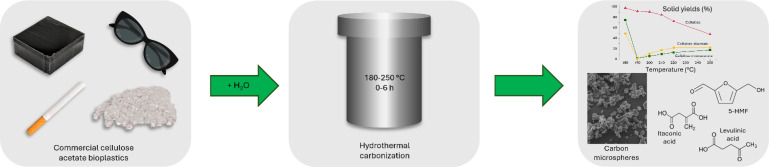

## Introduction

Plastic consumption has been escalating at an astonishing rate of 4 % per year, surpassing 380 million tons in 2022, with approximately 80 % of discarded plastics finding their way into landfills or natural environments. The negative repercussions are various, encompassing the release of micro and nanoplastics, chemical leaching, health hazards, resource depletion, greenhouse gas emissions, and habitat destruction.^[2]^ In this urgent situation, it is crucial to pivot towards a circular plastic economy and bioplastics stand out as one of the key strategies.^[3]^

Bioplastics can derive from renewable materials (bio‐based), be biodegradable, or possess both characteristics.[^3^, ^4^] They hold several potentials, such as bolstering circularity, providing biodegradation as an alternative end‐of‐life strategy, and substituting conventional fossil‐derived resources.^[5]^ Currently, their production remains niche, constituting around 1 % of the current plastic market, but forecasts indicate a rapid growth trajectory, projecting a significant increment from the current 2.2–6.3 million tons by 2027.^[6]^ Despite the enthusiasm surrounding bioplastics, they face challenges akin to conventional plastics, demanding thorough scrutiny to verify their effective eco–friendliness.^[3]^ Among the thorny topics around bioplastics, their end‐of‐life stands out, highly impacting their effective sustainability. Currently, waste management strategies for biodegradable bioplastics envision either treatment with organic waste (anaerobic digestion and/or composting) or with traditional plastics (recycling, incineration, and landfilling).^[7]^ However, the strategies designed for traditional plastics may prove unsuitable for biodegradable materials, which instead require specific disposal routes with relatively established conditions.^[8]^ Therefore, the disposal of bioplastics requires peculiar care to avoid management problems. For example, their treatment with organic wastes often results in incomplete degradation and contaminated digestate or compost.[^8^, ^9^, ^10^] Conversely, within landfills (the predominant destination for waste plastics, receiving approximately 50 % of global plastic waste),^[11]^ bioplastics’ degradation contributes to methane emissions, accounting for up to 80 % of greenhouse gas emissions during their end‐of‐life phase.^[7]^ Hence, there is room for research to explore solutions and propose pathways that could lead to more favorable and virtuous scenarios.

Hydrothermal carbonization (HTC) has raised attention in the framework of end‐of‐life strategies thanks to its potential for green and sustainable processes. In liquid water at 180–250 °C, HTC converts organic feedstocks into a carbon‐rich material (called hydrochar) and compounds dissolved into the liquid phase, both potential precursors for valuable products like advanced carbons and chemicals. HTC offers unique process advantages: mild conditions, no need for chemicals, suitability for highly wet substrates, and flexibility.^[12]^ Until now, HTC has been applied mainly to biomass‐based feedstocks, like organic wastes, algae, and lignocellulosic feedstocks. Applications to plastics are limited to few studies and typically include their conversion with a biomass fraction, added to favor their degradation.^[13]^ For example, HTC was effectively demonstrated to improve the disintegration efficiency of PET microplastics in sludge^[14]^ or to help dehalogenation of municipal solid wastes.^[15]^ Harsher hydrothermal conditions (in the liquefaction region) were demonstrated to be effective in converting waste plastics, such as polycarbonate and polypropylene, into a high‐energy density biocrude or chemicals.[^16^, ^17^] Bioplastics, either biodegradable or bio‐based, seem a potential feedstock for HTC. The current literature on HTC as an end‐of‐life strategy for waste bioplastics is very limited but with great potential, as shown by the recent works of Bracciale et al.^[18]^ and Marchelli et al..^[19]^

In this context, this study aims to explore new methods for residual bioplastic treatment, focusing on investigating the viability of HTC as an end‐of‐life solution for a specific bioplastic class: cellulose acetate. Cellulose acetate is a well‐established historical bioplastic with a market size of 5.1 billion USD (in 2021) and numerous applications, including cigarette filters, eyewear, protective films, membranes, and photographic equipment.[^20^, ^21^] It is a polysaccharide ester produced from the partial or complete acetylation of cellulose, mainly extracted from cotton linters or wood pulp.^[22]^ To improve its processability or physical properties, cellulose acetate is often added with low molecular weight plasticizers or modified in its chemical structure (for example, functionalized). These modifications have been reported to add complexity to its biodegradability, although the effects are unclear and should be studied more in‐depth. Cellulose acetate biodegradability rate is indeed debated: different researchers found contradictory results depending on the chosen degradation environment, the degree of acetylation of the polymer, and the presence of additives.^[10]^ Furthermore, cellulose acetate occupies a highly specialized niche, facilitating the separate collection of its waste, which, in turn, allows for a tailored disposal route rather than inefficiently collecting it with organic waste.^[8]^ Processes such as HTC could potentially serve as an alternative pathway for the valorization of this substrate in a biorefinery framework, thanks to their flexibility and capacity to synthesize advanced solid materials and platform chemicals, offering tailored options that dry thermochemical processes cannot attain.

This work systematically investigates the HTC of several cellulose acetates, in both pure and commercial forms. HTC was tested in a range of temperatures (from 180–250 °C) and residence times (from 0–6 h), with analyses on both the solid and liquid phases, to provide chemical insights behind the decomposition. Indeed, the reaction environment created by cellulose acetate, coupled with the additives, is unique and makes the material′s behavior highly depart from that of common (ligno)cellulosic substrates. Data were used to develop a reaction kinetics model characterized by a reaction order higher than 1 and accounting for all the species detected via analyses. Overall, this investigation underscores the potential effectiveness of HTC in valorizing cellulose acetates, positioning it as a viable strategy for their disposal and conversion into valuable products.

## Experimental Section

### Starting Materials

The two commercial bioplastics are cellulose diacetate (CD) and cellulose monoacetate (CM) from two companies in Northern Italy eyewear sector. Table 1 reports technical details of the products and Figure S1 the pictures of the CD and CM samples used in the investigation.

**Table 1:** Details of the two commercial cellulose acetates. T_m_ is the melting temperature, T_s_ is the softening temperature. * indicates data from technical datasheets.

Name	Origin	Appearance	Tm* (°C)	Ts* (°C)	Density* (g/cm3)	Additive (wt. %)
Cellulose diacetate (CD)	Wood pulp and cotton wool	Blocks 2x2x0.5 cm	>180	88–107	1.27	Acetyl triethyl citrate (40 %)
Cellulose monoacetate (CM)	Hemp	Spheres 3 mm diameter	>180	100	1.27	Triethyl citrate (47 %)

CD and CM theoretically differ in the numbers of acetyl groups substituting hydroxyl groups of the cellulosic backbone: 2 in the case of CD and 1 for CM, occupying a weight percentage of 34.9 and 21.1 % of the starting material, respectively.^[23]^ They contain additives (acetyl triethyl citrate in CD and triethyl citrate in CM) used to improve the processability of the materials, decreasing the glass transition temperature and broadening the temperature processing window.^[24]^ The employed additives are recognized as “green” since they derive from renewable sources.^[25]^

Pure triethyl citrate, acetyl triethyl citrate, and cellulose as a microcrystalline powder were purchased by Sigma Aldrich. CD was further purified from the plasticizer to constitute a reference material (CD‐pure) through a re‐precipitation method. Specifically, 20 g of CD were introduced in a 1 L round‐bottom flask equipped with magnetic stirring and dissolved in 400 mL of acetone. The resulting solution was precipitated in 600 mL of hexane producing the purified material as a white precipitate. The mixture was filtered and the precipitate was washed by 2 portions of hexane (100 mL each) and dried in an oven at 50 °C overnight. To achieve the complete elimination of the additives, the precipitation procedure was performed twice. The procedure allowed to separate CD‐pure (>99 % pure) as a white powder, and the additive (dense light‐yellow liquid) with a 96 % yield.

### HTC Runs

HTC runs were performed in a batch 50 mL stainless steel reactor.^[26]^ Without any pre‐treatment, 3.5 g of material were placed into the HTC reactor with distilled water at a constant mass ratio (B/W) of 0.125, a value typical of HTC^[27]^ and that ensured that the solids were entirely submerged.

The reactor was then closed and flushed three times with pure nitrogen, creating inert and atmospheric pressure initial conditions for the gas phase. Afterwards, it was heated through an external electric resistance (heating time of 20–30 min, depending on the set temperature). Once the residence time (corresponding to the constant temperature phase) had passed, the reactor was quickly cooled by soaking it in cold water (reaching a temperature lower than 50 °C within 5 min). The produced gas was let flow in a vertical cylinder filled with water and closed at the top, allowing the measurement of its volume. The gas mass was calculated from its volume via the ideal gas law, assuming for simplicity its composition to be 100 % CO_2_.^[28]^ The reactor was finally opened, and its content vacuum filtered through a 0.45 μm filter membrane (Whatman). The recovered solid was dried at 105 °C overnight and then weighed.

The solid (SY) and gas (GY) yields were obtained by dividing their amount by the initial mass of the sample, while the liquid yield (LY) was obtained as the complement of solid and gas yields.

The investigated operating conditions are as follows. Commercial CD and CM were hydrothermally treated for 1 h and 180, 190, 200, 210, 220, and 250 °C; the kinetics of commercial CD at 210 °C and different residence times of 0, 1/4, 1/2, 1, 3, and 6 h; separate runs on CD‐pure and additives for 1 h and 180, 190, 200, 210, 220, 250 °C. As reference runs, pure cellulose was tested both alone and together with acetic acid (glacial, purity≥99 %) at an acetic acid/cellulose mass ratio of 0.74, mimicking the cellulose diacetate composition, at 1 h and temperatures of 180, 190, 200, 210, 220, 250 °C. To facilitate a comparison between values, for CD‐pure and cellulose plus acetic acid, SY was computed as if the plasticizer (assumed to constitute 40 % in mass of the starting material) was present. Each experimental condition was tested at least twice, and standard deviations were reported.

### Solid Materials Characterization

Bioplastics and hydrochars were characterized through various analytical techniques.

Elemental composition was determined through a LECO 628 Elemental Analyzer, in accordance with ASTM D‐5375 for carbon (C), hydrogen (H), and nitrogen (N). The oxygen (O) content was determined by the difference with C, H, N, and ash. The ash content was assessed by placing samples in a muffle at 550 °C for 6 h.

The morphology was investigated through JEOL JSM‐7001F Field Emission Scanning Electron Microscopy (SEM) at an electron beam energy of 5–15 keV and working distance between 8 and 10 mm. Samples were gold‐coated before the analyses.

Fourier Transform InfraRed (FT‐IR) spectra were collected on different specimens in transmittance configuration; solid solutions of the powdered samples, mixed with KBr, were pressed in order to obtain pellets, which were analyzed by means of a Nicolet Avatar 330 (Thermo Fischer) FT‐IR spectrophotometer. The following scan conditions were adopted: wavenumber range: 4000–400 cm^−1^, number of scans: 64, resolution: 4 cm^−1^.

### Liquid Phase Characterization

The HTC liquid phase obtained after the filtration was characterized in terms of composition, total organic carbon (TOC) and pH.

TOC was measured through a FORMACS HT‐iTOC analyzer (Skalar Analytical B.V., Netherlands) according to the ASTM D7573 standard. Each measurement was performed at least three times and the average value reported.

Nuclear Magnetic Resonance (NMR) analysis was performed on a Bruker Biospin Avance spectrometer equipped with a Broadband Inverse probe and operating at a proton frequency of 400.13 MHz. The ^1^H‐NMR analysis exploited a zgpr pulse sequence with water pre‐saturation. The measures were carried out with a spectral width of 11 ppm (−1–10 ppm), a relaxation delay of 10 s, an acquisition time of 6.8 s. According to a previously optimized method,^[26]^ the samples were prepared by diluting 0.2 mL of HTC liquor with 0.4 mL of D_2_O containing 0.05 % w/w of 3‐(trimethylsilyl) propionic‐2,2,3,3‐d4 acid sodium salt (TSP). TSP was used both as a reference standard (δH=0.00 ppm) and as a calibration standard (C(STP)=3.2 mM).

The mass yields of compounds dissolved into the liquid phase were computed from NMR concentrations and accounted for the mass increment of the liquid phase through the liquid yield. Thus, values were converted to the ratios between the mass of the compound (or of the organic carbon) and the initial mass of the treated sample, employing Equation 1:

(1) $$\frac{m_{i,liq}}{m_{0}}=\frac{C_{i,liq}}{\rho_{liq}}\left(\frac{1}{B/W}+LY\right)$$

in which m_i,liq_ is the mass of dissolved i‐compound into the liquid phase, m_0_ is the initial mass of the treated sample, C_i,liq_ is the mass concentration of dissolved i‐compounds (from NMR), and ρ_liq_ is the density of the liquid phase (assumed to be constant and equal to that of water, 1000 g/L).

A theoretical value of total organic carbon (TOC_NMR_) was computed by summing the contribution in carbon of every compound dissolved into the liquid phase as measured via NMR.

### Reaction Kinetics Model

**Figure 1:**
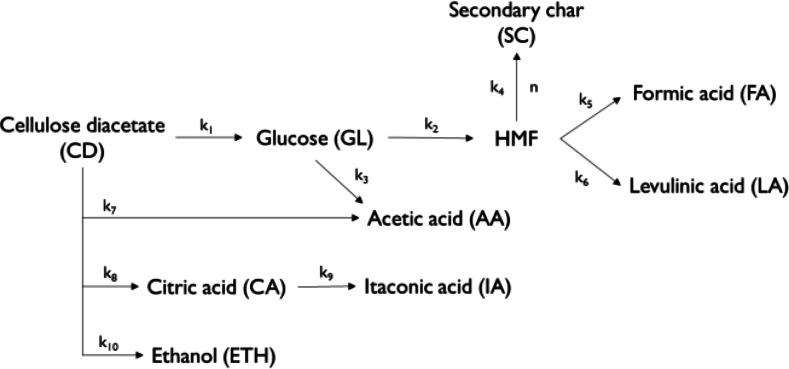
Reaction scheme used for the lumped kinetic model for the HTC of CD.

A lumped kinetic model was developed to compute reaction rate constants (k_i_) following the scheme reported in Figure 1, based on the tests performed on CD at 210 °C and different reaction times. The hydrochar was considered to be composed of residual CD and secondary char (SC). The gas phase was neglected. All reactions were assumed to be first order apart from the 5–hydroxymethylfurfural (HMF) repolymerization into SC, assumed of variable order (set at 1, 1.5, or 2).

The rate constants k_i_ were computed for the different values of n by solving the system of differential equations (ODEs) Equations (2–11), following a resolution method presented in a previous work.^[26]^ It consists of a numerical method where k_i_ are evaluated by combining a multi‐step algorithm with a minimization function, represented by the Python functions ‘odeint’ and the basin‐hopping attraction algorithm ‘basinhopping’, respectively. The minimized error function E(k,n) is defined as the squared sum of the differences between the experimental (C^exp^) and predicted (C^pred^) concentrations at different times (Equation 12.

(2) $$\frac{d\left[CD\right]}{dt}=-k_{1}\left[CD\right]-k_{7}\left[CD\right]-k_{8}\left[CD\right]-k_{10}\left[CD\right]$$

(3) $$\frac{d\left[GL\right]}{dt}=k_{1}\left[CD\right]-k_{2}\left[GL\right]-k_{3}\left[GL\right]$$

(4) $$\frac{d\left[HMF\right]}{dt}=k_{2}\left[GL\right]-{k_{4}\left[HMF\right]}^{n}-k_{5}\left[HMF\right]-k_{6}\left[HMF\right]$$

(5) $$\frac{d\left[SC\right]}{dt}={k_{4}\left[HMF\right]}^{n}$$

(6) $$\frac{d\left[FA\right]}{dt}=k_{5}\left[HMF\right]$$

(7) $$\frac{d\left[LA\right]}{dt}=k_{6}\left[HMF\right]$$

(8) $$\frac{d\left[AA\right]}{dt}=k_{7}\left[CD\right]+k_{3}\left[GL\right]$$

(9) $$\frac{d\left[CA\right]}{dt}=k_{8}\left[CD\right]-k_{9}\left[CA\right]$$

(10) $$\frac{d\left[IA\right]}{dt}=k_{9}\left[CA\right]$$

(11) $$\frac{d\left[ETH\right]}{dt}=k_{10}\left[CD\right]$$

(12) $$E\left(k,n\right)=\sum_{i}{\left(C_{i}^{exp}-C_{i}^{pred}\right)}^{2}$$

## Results and Discussion

### HTC of Cellulose Acetates

**Figure 2:**
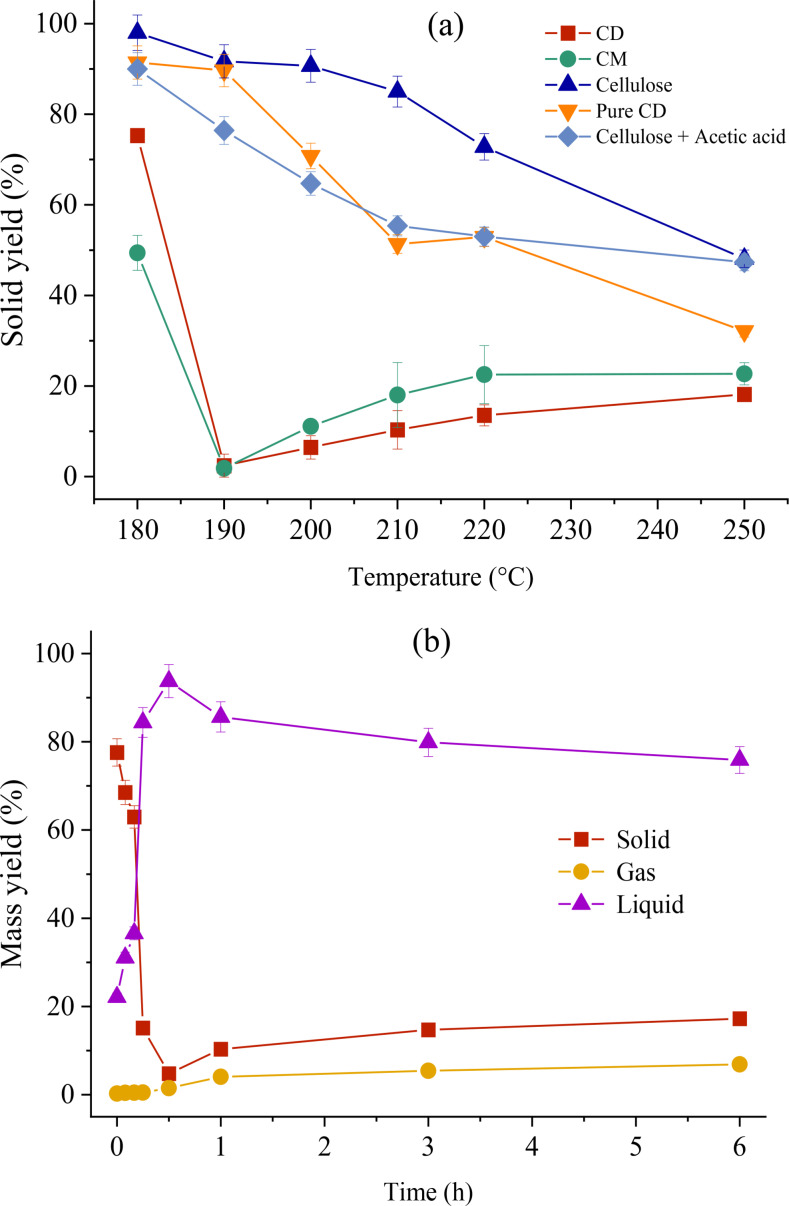
(a) Effect of temperature on solid yields of commercial CD and CM, pure CD, pure cellulose, and cellulose plus acetic acid (1 h); (b) effect of time on the mass yields of commercial CD, at 210 °C. The lines connecting datapoints only have the purpose of facilitating trend visualization.

The trends of mass yields and composition with HTC temperature and time (reported in Figure 2, Table 2 and Figure S2) show the peculiar behavior of commercial cellulose acetates. These patterns diverge from those typically observed in common lignocellulosic biomasses (which show a progressive dissolution with severity^[29]^) as well in other commercial cellulose acetates (which only tend to dissolve in the liquid and at harsher conditions).^[18]^ Indeed, SYs exhibit a distinctive downward‐upward trend, with a minimum point near zero (observed at 190 °C and 1 h and at 210 °C and 30 min) that becomes a threshold dividing two conversion stages. Interestingly, this minimum point is observed at 20 °C more if tap water is employed (tests with tap water had been conducted in a previous experimental campaign^[30]^ and are not reported here). Before the minimum, the trend is downward and consists of a dissolution stage, starting from the release of additives from the parent material. After the minimum, SY increases with temperature, with a secondary conversion involving the formation of a char via liquid‐solid reactions. This behavior agrees with the distribution of carbon among the different phases (Figure S2), where it is evident how carbon shifts from the solid to the liquid and vice versa as the treatment severity increases. For the sake of clarity, mass yield values also at residence times of 5 and 10 minutes (Figure 2b) are also reported.

**Table 2:** Details of HTC of cellulose acetates at 1 h: mass yields, elemental composition, O/C and H/C atomic ratios of hydrochars, TOC and pH of liquors. Ash equals zero for all the samples. Standard deviations <2.5 % for mass yields and <1 % for ultimate composition. Oxygen was computed by difference.

Sample		Mass yields (%)			Ultimate analysis (wt. %)				O/C	H/C	TOC (g/L)	pH
		Solid	Gas	Liquid	C	H	N	O				
CD	Raw	–	–	–	48.9	6.2	0.0	44.9	0.7	1.5	–	–
	180 °C	75.3	0.2	24.4	45.7	6.3	0.1	47.9	0.8	1.7	11.6	2.6
	190 °C	2.4	0.8	96.8	–	–	–	–	–	–	50.1	
	200 °C	6.5	1.2	92.3	–	–	–	–	–	–	48.5	
	210 °C	10.3	4.1	85.6	66.0	5.5	0.1	28.4	0.3	1.0	41.9	2.2
	220 °C	13.5	4.4	82.1	65.1	5.0	0.1	29.8	0.3	0.9	34.4	2.1
	250 °C	18.1	5.6	76.2	67.4	5.0	0.1	27.4	0.3	0.9	35.9	
CM	Raw	–	–	–	48.8	6.5	0.0	44.7	0.7	1.6	–	–
	180 °C	49.4	0.2	50.4	46.8	6.4	0.0	46.8	0.8	1.7	26.0	2.4
	190 °C	1.8	0.7	97.5	–	–	–	–	–	–	53.6	
	200 °C	11.1	2.9	86.0	65.2	5.2	0.1	29.5	0.3	1.0	43.9	2.8
	220 °C	22.5	6.2	71.3	66.3	5.0	0.1	28.6	0.3	0.9	36.6	2.3
	250 °C	22.7	8.6	68.7	66.4	5.0	0.1	28.5	0.3	0.9	33.2	

The first stage (before the minimum) consists of the initial material decomposition involving the partial release of the additive, leading at 180 °C to SYs of 75.5 and 49.4 % for CD and CM, respectively. The material shrinks and loses transparency: at this stage, the hydrothermal conversion consists of the material hydrolysis with the release of additives, as confirmed by liquid data later discussed. The presence of additives is crucial compared to pure substrates, as they impart entirely different properties to the commercial material. They make up 40 and 47 % of the material′s weight and possess higher mobility and lower thermal stability than the cellulose backbone.^[31]^ Therefore, they can be released from the material at milder conditions, leading to almost complete dissolution. The decrease in SY is accompanied by a carbon migration from the solid to the liquid phase: approximately 20 % of the carbon present in CD and 40 % in CM transitions into the liquid phase, subsequently increasing its TOC (Figure S2). Consequently, the resulting hydrochars exhibit a composition only slightly different from the initial material, with carbon contents of 45.7 and 46.8 %, vs the initial 48.9 and 48.8 %. Hence, the material does not undergo any carbon‐concentration mechanisms, leading to an increased O/C and H/C ratios instead of an opposite trend. The resulting hydrochar can thus be likely intended as the initial material largely deprived of plasticizers, released into the liquid phase. The difference in SY between CM and CD likely stems from the plasticizer itself and the dimension of the samples. Indeed, triethyl citrate in CM possesses a less complex chemical structure than triethyl acetyl citrate in CD and the starting materials are characterized by significantly different surface areas, much higher in CM in respect to CD, Figure S1.

After 1 h (at 190 °C), CD and CM reach their minimum SY of 1.8 and 2.4 %, respectively. Simultaneously, the liquor TOC peaks at its maximum (50.1–53.6 g/L), while gas production remains negligible (<1 %). This behavior indicates a near‐complete material dissolution, with the plasticizer fully released and the cellulose acetate structure hydrolyzed. The temperature and the unique liquid environment resulting from the dissolution likely induce the cleavage of acetyl groups and glycosidic bonds within the acetate structure. As a result, the liquid phase begins to consist of additives and cellulose derivatives, as discussed later. The role of additives appears pivotal in this context, both chemically and structurally. Indeed, their early release generates compounds such as citric acid and acetic acid, reducing the reaction environment′s pH (down to 2.1) and catalyzing HTC. They also liberate ethanol, which contributes to modifying the chemistry of the hydrothermal environment by lowering the polarity of the medium.

In the second stage (beyond the minimum), SY progressively increases with the process severity, reaching values of 18.1–22.7 %. This trend is characteristic of secondary char formation during HTC.^[32]^ The SY increment, coupled with HMF consumption and the detected spherical morphology as later discussed, confirms its presence: HMF in the liquid phase undergoes repolymerization, resulting in a material composed of nano/microspheres, i. e. the secondary char (refer to Section 3.4, Figure 5a, and Figure S3). Simultaneously, the TOC decreases with temperature (from values of 50–53 down to 33–36 g/L), confirming the transfer of carbon from the liquid to the solid phase. A carbon–rich hydrochar forms, with C content ranging from 65.1–67.4 %. The O/C and H/C atomic ratios decrease compared to the starting materials. Despite variations in temperature, hydrochar properties exhibit only minor changes, indicating a slight rearrangement of the material with severity.

In summary, under all conditions, the behaviors of CD and CM remain similar, exhibiting slight differences in yields and hydrochar composition, which reflect the minor discrepancies in the initial material compositions. Once the additive is released from the matrix, the cellulose backbone is left behind, and its further cleavage leads to glucose derivatives that become precursors for secondary char in the form of carbon microspheres. Secondary char forms easily from the starting substrates following a pathway similar to that of pure sugars–a positive observation for its recovery and potential for further valorization for advanced carbons.^[33]^ Conversely, the behavior of cellulose acetates significantly diverges from that of common lignocellulosic biomasses and cellulose itself. Indeed, these substrates undergo a predominance of bulk reactions (forming a primary char akin to the parent biomass) compared to repolymerization reactions (yielding secondary char) due to the lower amount of secondary char precursor.^[29]^ In this regard, additive derivatives are pivotal for the entire dissolution of cellulose acetates.

### Liquid Phase Composition

**Figure 3:**
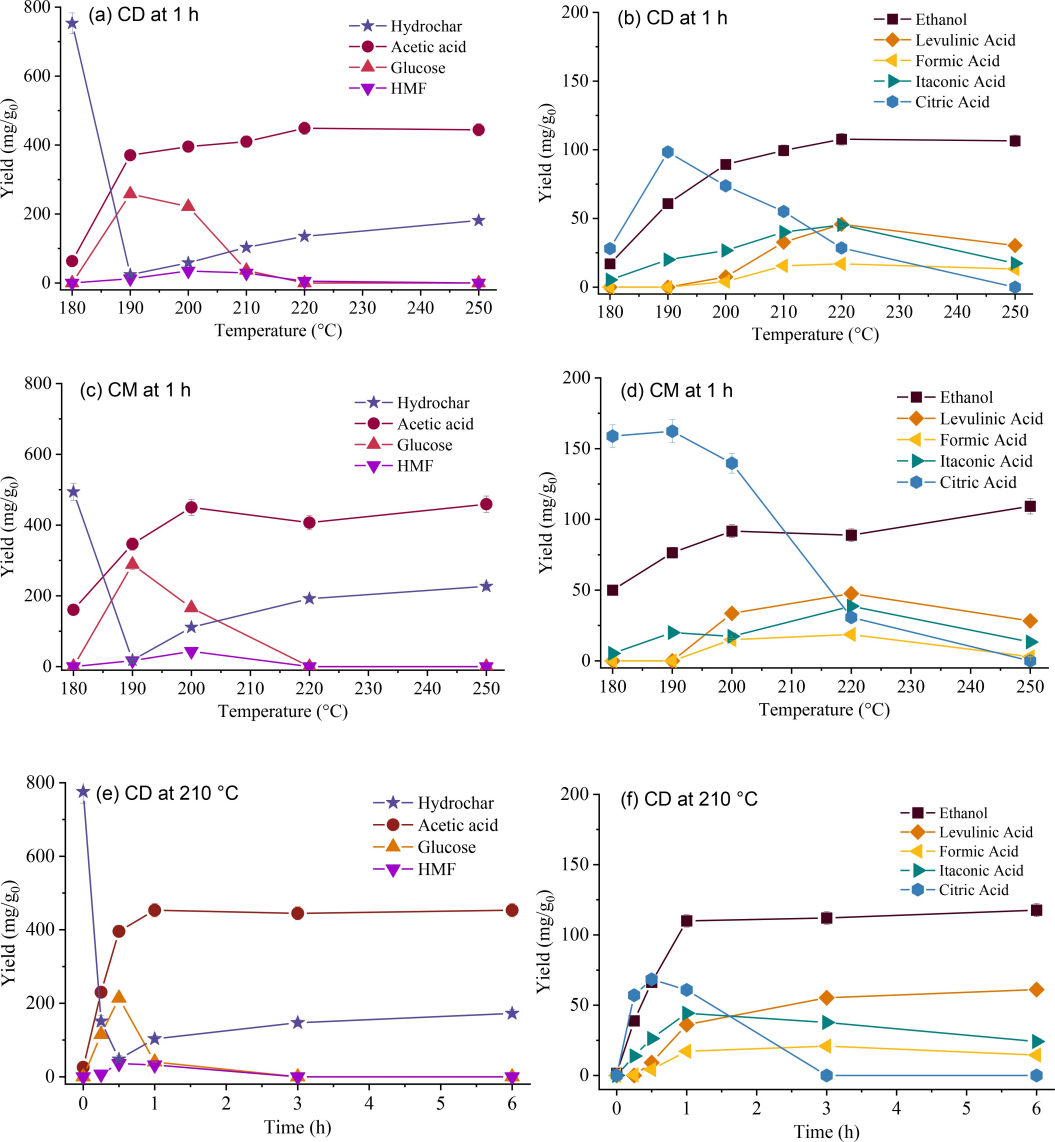
Mass yields of hydrochars and compounds detected into the liquid phase deriving from HTC of commercial CD and CM at different temperatures (a–d) and time (e–f). Data are expressed as mg of *i*‐compound per gram of starting material. Error bars represent standard deviations; where not visible, they are negligible. The lines connecting datapoints only have the purpose of facilitating trend visualization.

Figure 3 shows the effect of temperature and time on the yields of compounds detected through NMR within the liquid phase (details on the spectra are reported in the Supplementary Materials). The variety of compounds reflects the peculiar nature of the cellulose acetates, with additives playing an important role. Indeed, in addition to the typical compounds expected from a cellulose derivative (like glucose, HMF, and carboxylic acids), we also detected ethanol, citric acid, and itaconic acid, which originate from the direct degradation of the ethyl citrate derivatives. Their presence highly modifies the reaction environment and makes it unique. Derivatives from CD and CM differ slightly, with CM having slightly higher yields of secondary char precursors (glucose, HMF) following solid yields. It is worth noticing that the TOC computed from NMR data is comparable with the measured one (Table S2), with only slight underestimations. This observation further confirms the reliability of the data, while the “missing carbon” (always lower that 10 %) could be attributed to some colloidal particles and/or oligosaccharides present in the liquid phase. Some of the aforementioned compounds were also identified by Bracciale et al.^[18]^ in their investigation on the HTC of cellulose acetates, although they did not report the concentration of all products.

The liquid environment is consistently acid, with a pH range of 2.1–2.8, primarily attributed to high acetic and citric acid yields. The pH values measured in the present study are markedly lower than those reported by Bracciale et al.,^[18]^ even though they operated at a higher B/W. Indeed, acetic acid stands out as the predominant chemical, presenting a concentration (0.9 M) nearly five times higher than the minimum required for achieving a pH of 2.4 (considering an acid dissociation constant of 4.76).^[34]^ Citric acid (0.1 M) and other acids further reduce pH. This acidic mixture significantly facilitates cellulose acetate conversion. The heightened concentration of H^+^ ions can effectively weaken hydrogen bonds within the cellulose chain and promote the cleavage of acetyl groups from the cellulose backbone. Acid‐assisted HTC is a common technique for catalyzing reactions and tuning hydrochar properties.[^35^, ^36^, ^37^] However, acetic acid is likely not the only compound favoring material conversion. Indeed, ethanol (highly present, up to 118 mg/g_0_) could behave as a co‐solvent with water, widening the range of polarities. Therefore, it seems that a synergistic action of several chemicals likely confers the material such a high dissolution rate, which has never been observed for other cellulose‐based materials.

More in detail, regarding the trends of single compounds, the following considerations can be drawn.

*Glucose* emerges in the early conversion stages. For both CD and CM cases, it reaches its maximum yield at 190 °C and 1 h or 210 °C and 30 min, progressively disappearing at higher temperatures and residence times. Glucose derives from the cleavage of glycosidic bonds of the cellulose backbone and, indeed, its maximum yields correspond to the maxima of liquid yields and TOC (Table 2). It is a reaction intermediate: once formed, it dehydrates to HMF and degrades yielding other compounds, like furfural derivatives and some acids.[^32^, ^33^] Its release from cellulose acetate occurs at lower severities than from pure cellulose (which undergoes a significant degradation at temperatures greater than 220 °C),^[38]^ likely favored by the combined action of the acidic environment and ethanol. CM shows a slightly higher maximum yield of glucose than CD, 323.9 vs 289.5 mg/g_0_, in agreement with the higher material degradation.

In concomitance with glucose, *HMF* forms. HMF is a well‐known compound formed during the HTC of sugar‐rich substrates and directly derives from the dehydration of glucose and fructose.^[39]^ The slight shift in time and temperature of HMF formation compared to glucose agrees with its reaction mechanism. Thanks to its aldehydic structure, HMF then becomes a direct reactant in the synthesis of secondary char, which forms from its back polymerization.^[33]^ Trends along temperature and time agree with its intermediate nature: it reaches a maximum yield at 200 °C (38.9 and 47.9 mg/g_0_ for CD and CM, respectively) and progressively decreases to zero. Interestingly, we notice a mass discrepancy between hydrochar (actually, secondary char) and HMF yield performing a simple mass balance, considering the theoretical polymerization reaction of HMF (HMF+3H_2_O → hydrochar+H_2_O) often used in the literature. In the case of CD, considering an HMF yield of 38.9 mg/g_0_, the theoretical mass balance leads to 55.5 mg/g_0_ of secondary char. Also, removing the residual material deriving from the first stage (24.2 mg/g_0_, measured at the minimum SY) from the final hydrochar, we obtain 157.2 mg/g_0_, almost three times the value from the theoretical computation. Some acids could be adsorbed on the hydrochar,^[38]^ but it does not seem enough to justify the mass gap. Thus, while HMF is certainly a secondary char precursor, other mechanisms probably participate in secondary char formation. Further research would be helpful to untangle this problem, which could provide some new insights into formation mechanisms.

NMR also highlights the presence of carboxylic acids deriving from HMF and sugar degradation. In particular, *acetic acid* occupies the highest fraction among the detected compounds, reaching yields of 494.9–498.6 mg/g_0_: around half of the material is converted to acetic acid. It mainly derives from the acetyl groups of cellulose acetate, whose cleavage is favored by the acidic environment induced by its self‐catalysis and additive derivatives.

A smaller fraction of acetic acid derives from the HMF oxidation^[40]^ and the hydrolysis of the acetyl groups of triethyl acetyl citrate, the plasticizer contained in CD. For both temperature and time variations, acetic acid yield tends to reach a plateau in concomitance with the exhaustion of primary kinetics. Then, *levulinic acid* and *formic acid* form from the rehydration of HMF above 200 °C. The slight decrease in levulinic acid can be due to its participation in hydrochar formation.^[39]^

*Ethanol*, *citric acid* and *itaconic acid* stem from the plasticizers. These compounds are typically not detected from the hydrothermal conversion of lignocellulosic biomass; indeed, their trends closely resemble those obtained in the tests with pure plasticizers, as later discussed. In light of this lack of previous knowledge, it is difficult to ascertain their role in the HTC environment, which possibly paves the way for future focused studies. Ethanol and citric acid, particularly, constitute a significant fraction and originate from the hydrolysis of ester bonds present in triethyl citrate and triethyl acetyl citrate. The yield of ethanol increases with temperature and time, with its maximum formation observed between 180–200 °C (at 1 h) and 0–60 min (at 210 °C). Beyond these conditions, it stabilizes to nearly identical yields of 118.7 mg/g_0_ for both CM and CD. Trends of citric acid demonstrate its nature of reaction intermediate. It peaks at 190 °C (at 1 hour) and at 30 min at 210 °C, decreasing close to a zero yield above 220 °C (Figures 3b and 3c) and after 180 minutes (Figure 3f). It probably ultimately converts to itaconic acid and CO_2_. At 190 °C, its yield from CM surpasses that from CD, potentially due to simpler dissolution from triethyl citrate, which undergoes more facile hydrolysis in the absence of acetyl groups. As discussed previously, citric acid aids in reducing the overall pH of the hydrothermal environment, thereby facilitating the conversion of cellulose acetate.^[35]^

*Itaconic acid*, a dicarboxylic acid rarely found in biomass hydrothermal conversion, likely derives from citric acid. Temperature and time trends show that it reaches its maximum at 220 °C and 60 min, with a peak yield of 49.9 mg/g_0_. It generally derives from the microbial conversion of sugars, which often relies on citric acid,^[41]^ probably its precursor in this work. Hydrothermal conditions could induce the loss of a carboxylic group and the dehydration of citric acid. Trends seem to confirm the supposition: as citric acid disappears, itaconic acid forms. The slight decrease in yield of itaconic acid at 250 °C could be attributed to a higher conversion towards CO_2_ (favored by temperature) and a shift in the thermodynamic equilibrium between citric and itaconic acid reactions. Concerning chemical recovery, itaconic acid holds substantial value as a building block for producing certain polymers and as an intermediate for methyl tetrahydrofuran–a nascent component in biofuel synthesis and an environmentally friendly solvent for chemical reactions.^[41]^ Hence, its separation could be scrutinized in a potential biorefinery development.

### Behavior of Pure Compounds

We also performed experiments on pure compounds to clarify the mechanisms behind reaction pathways. CD‐pure, pure cellulose plus acetic acid (kept at the acetic acid/cellulose ratio of 0.74 characteristic of CD), and pure cellulose.

The *CD‐pure* consists of the commercial CD deprived of the plasticizer. The main difference with the behavior of the original CD is that it does not show any local SY minima: at 180–190 °C pure CD undergoes only a slight mass loss, which progressively increases with temperature reaching a SY of 32.2 % at 250 °C (Figure 2a). The liquid phase consists exclusively of acetic acid and HMF, confirming that citric acid, ethanol, and itaconic acid derive exclusively from the plasticizer. The maximum HMF yield is 64.6 mg/g_0_, corresponding to a value of 38.7 mg/g_0_ if we account for a fictitious 40 % additional mass in the initial feedstock, corresponding to the plasticizer (which does not actively contribute to HMF formation). This value is very similar to the 34.6 mg/g_0_ from the commercial CD, suggesting that this yield is probably the maximum achievable from this precursor under the investigated conditions. The HMF production begins at a higher temperature for pure CD than commercial CD (210 vs 190 °C), likely due to the catalytic effect of the plasticizer. Acetic acid predominates inside the liquid phase and grows progressively with the temperature. It reaches a maximum yield of 508 mg/g_0_ (or 304.8 mg/g_0_ if referred to as a pure CD plus plasticizer) to be compared to 494.9 mg/g_0_ for the commercial one: acetyl groups inside the additive certainly contribute to the mass increment and also to the dissolution.

*Pure cellulose plus acetic acid*, like CD‐pure, shows no SY minima but a SY progressively decreasing with temperature: the pH reduction alone is insufficient to cause the material′s almost total dissolution into the liquid phase. Compared to CD‐pure, cellulose plus acetic acid showcases lower SY at low temperatures, similar SY at intermediate temperatures, and a reverse trend at 250 °C (32.1 % for CD‐pure vs. 47.3 % for cellulose+acetic acid, Figure 2a). This behavior is probably due to the different structure of the substrates. For CD, acetic acid is embedded inside the solid phase, requiring a deacetylation step before being released into the liquid phase. For cellulose plus acetic acid, this last is already dissolved into the liquid phase and, therefore, immediately available to the solid material, favoring the degradation/depolymerization reaction kinetics at low temperature. Besides, the comparison with *pure cellulose* confirms the strength of acetic acid in reducing the temperature necessary for the conversion: at 200 °C, the SY is 90.7 % for cellulose and 64.7 % with the addition of acetic acid. At this temperature, acetic acid likely acts as a catalyst, lowering the pH and promoting cellulose hydrolysis and the formation of precursors for secondary char. At 250 °C, cellulose reaches the same SY as cellulose combined with acetic acid (with 48.1 %). It is known that this condition is sufficient to induce cellulose hydrolysis and carbonization, as well as the formation of secondary char.^[38]^ Under these conditions, the severity of the process predominates over the acidity imparted by acetic acid. Despite enhancing the conversion, acetic acid does not appear to be the primary factor influencing the SY minimum observed in CD and CM (Figure 2a), where the initial conversion is driven by the additives.

**Figure 4:**
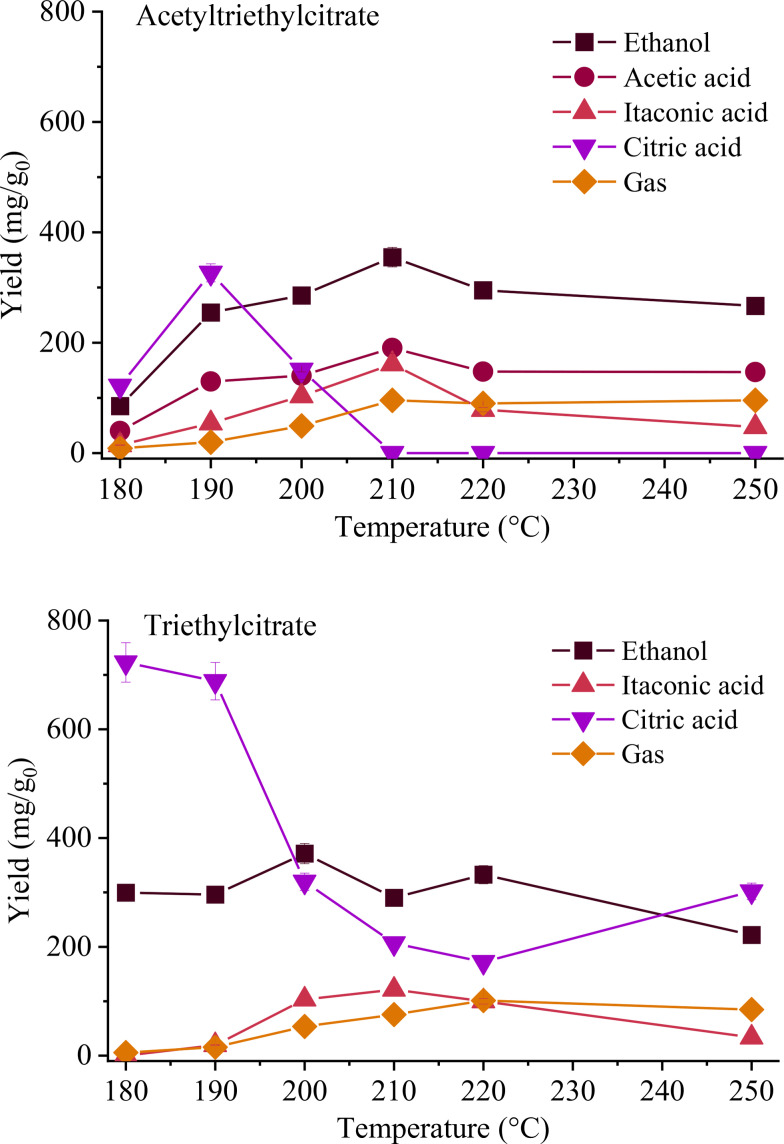
Yields of compounds dissolved into the liquid phase from the HTC of additives at different temperatures and 1 h. Gas is also reported. Data are expressed as mg of *i*‐compound per gram of starting material. The lines connecting datapoints only have the purpose of facilitating trend visualization.

As part of the baseline runs, we investigated the HTC of *pure additives–*triethyl citrate and triethyl acetyl citrate (with Figure 4 depicting the yields of dissolved compounds). These additives are esters derived from citric acid, featuring a citric backbone to which three ethyl groups are attached, with triethyl acetyl citrate additionally incorporating an acetyl group. Their conversion initiates at 180 °C, aligning consistently with observations from commercial cellulose acetates. Notably, neither of these two additives yields solid products but consistently produces a gas phase, whose yield never exceeds 10 %. Their derivatives differ in the presence or absence of acetic acid, which mainly originates from the acetyl groups. However, both plasticizers lead to the formation of citric acid, ethanol, and itaconic acid. In both instances, ethanol represents the major component, reaching yields up to 355.1 mg/g_0_. It arises from the hydrolysis of ethyl groups, with its slight decrease at high temperatures likely attributed to its enhanced conversion to CO_2_. Notably, the maximum yield of citric acid is roughly double for triethyl citrate compared to acetyl triethyl citrate (723.0 vs 326.6 mg/g_0_), likely due to former′s higher content of citrate ester groups. The degradation of citric acid to itaconic acid is supported by their complementary trends with temperature.

In conclusion, the comparison between CD and pure substrates highlights the crucial role of the plasticizer additive. It constitutes a notable mass percentage (40 %), contributing significantly to the overall mass loss. Its derivatives induce a rapid decrease in pH, thereby catalyzing reactions. Moreover, it also impacts the material structurally: once released, it leaves behind a highly porous structure with a greater internal surface area, making it more easily degradable than the pure material itself.

### Properties of Hydrochars

Results show cellulose acetates undergo a deep re‐organization as the HTC severity increases. As a result, hydrochar properties highly depend on the production stage, with properties resembling those of the starting material in the first stage and those of a typical secondary char in the second stage.

**Figure 5:**
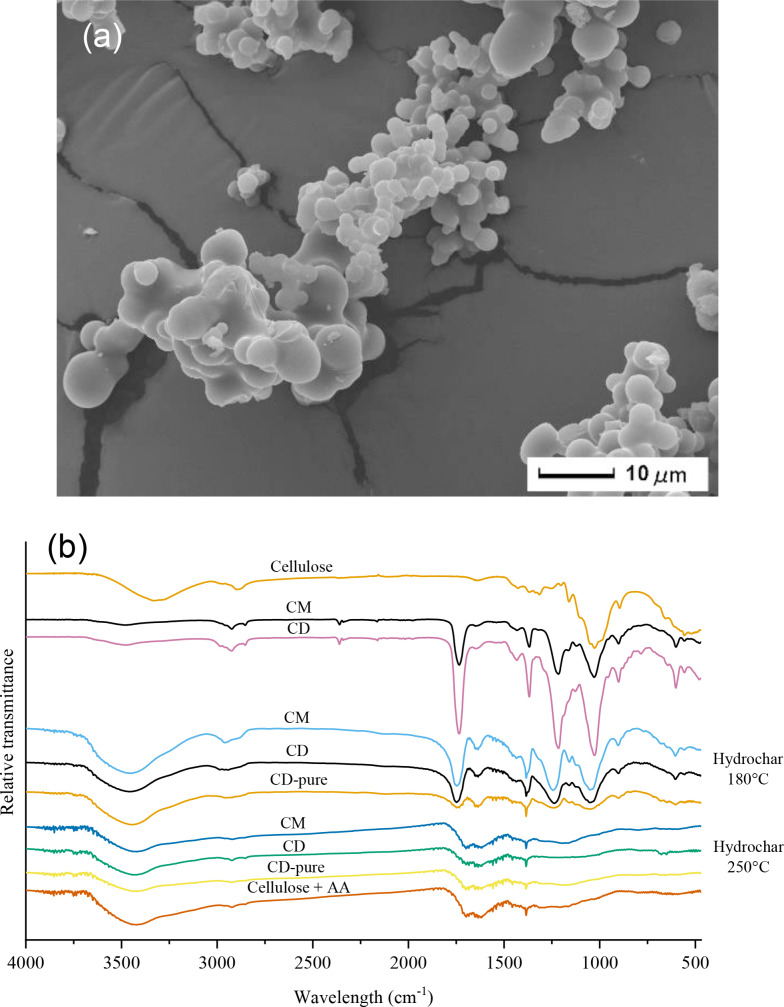
(a) SEM image of hydrochar from CM at 250 °C 1 h; (b) FTIR spectra of cellulose, commercial CM and CD and hydrochars produced at 180 and 250 °C, at 1 h of residence time.

The hydrochars produced in the second stage exhibit a spherical morphology (depicted in Figure 5a and Figure S3), supporting the hypothesis that they originate nearly completely from secondary char formation. This morphology is commonly found in HTC, especially in materials rich in saccharides.^[42]^ In our case, spheres do not have a perfectly formed shape, and the particle size distribution is wide, ranging between 0.5 and 2 μm, probably due to the heterogeneity of the environment itself and the starting material. However, tuning operating conditions (like temperature, time, and pH) could produce more uniform and well‐defined shapes with a narrower size distribution, making secondary char a valuable product that could be valorized for further applications.

In terms of composition, the hydrochar originating from the initial stage closely resembles the starting material. Conversely, the hydrochar resulting from the second stage has a higher carbon content (ranging from 65.1–67.4 %, Table 2) and a reduced oxygen content (decreasing from 44.7–44.9 to 27.4–28.5 %). It is worth noticing that compared to secondary char derived from glucose or sugar derivatives,[^26^, ^39^, ^40^] the O/C and H/C ratios exhibit remarkable similarities, standing at 0.3 and 0.9, respectively. Slight variations observed with severity suggest that the secondary char undergoes only minimal re‐arrangements once is formed.

Similarly, FTIR spectra (depicted in Figure 5b) highlight differences in surface properties between the first and second stages. Hydrochars produced at 180 °C from cellulose acetates resemble the original bioplastics. CM and CD spectra almost overlap, while pure CD has a less intense spectrum, probably due to the absence of additives, which directly affect the material structure, with an increase in the variability of the chemical sites related to the present functional groups. In particular, 180 °C hydrochars show 1020–1050 cm^−1^ and 1230 cm^−1^ bands corresponding to C−O stretch related to acetate groups in cellulose acetate; the bending of 1370–1380 cm^−1^ due to methyl groups of the acetyl units (−CH3CO); the vibration of carbonyl groups C=O at 1735 cm^−1^ present in the acetyl (CH_3_CO−) units introduced during the acetylation process; a broad band, centered at 2920 cm^−1^, due to the stretching vibrational modes of C−H groups; a wide 3000–3700 cm^−1^ band due to −OH stretching of hydroxyl groups. The −OH band is quite absent in the starting material, probably due to the acetylation process, which replaces −OH groups of cellulose with acetate groups, resulting in a decrement/removal of the −OH band. Conversely, the hydrochars produced at 250 °C show a very different spectrum. The only peaks in common with the original material and 180 °C hydrochars are 1370–1380 cm^−1^, due to the presence of methyl, methylene and acetyl groups, plus the evidence of the −OH band. Hydrochars show new bands at 1620 and 1690 cm^−1^. The 1620 cm^−1^ signal appears as an overlapping of different contributions, attributable both to aromatic C=C stretching and to water adsorbed into the sample surfaces. Even if the carbon is mainly amorphous inside the solid, some short‐range aromatic or conjugated carbon structures could give rise to the first contribution of the band.^[43]^ The 1690 cm^−1^ could be attributed to the C=O stretching of carbonyl groups linked to oxygen‐containing groups like carboxylic acids and aldehydes. As for the composition, there are no significant differences between the hydrochars from pure CD and commercial acetates, nor with cellulose plus acetic acid. Overall, the FTIR spectra of the secondary char are very similar with those deriving from pure fructose and glucose.[^39^, ^44^, ^45^]

The observations above show that the final secondary char, comprising almost 100 % of the final hydrochar, has morphology, composition and surface properties similar to carbon nano/microspheres deriving from saccharide‐rich substrates. Thanks to their well‐defined spherical morphology and richness in functional groups, these nano/microspheres represent, in all respects, a significant field of research as precursors for the synthesis of advanced carbon materials across various applications, including electrodes, gas capture, electrocatalysis, and adsorption.[^32^, ^46^, ^47^] Results indicate that even materials that appear dissimilar to biomasses, such as cellulose acetate, can yield to around 18.1–22.7 % of carbon nano/microspheres. Additionally, these spheres are produced with high purity, eliminating the need for a removal stage from the main substrate–an advantage not typically found with lignocellulosic biomasses.^[48]^ This outcome presents a promising perspective within the context of a biorefinery, envisioning the synthesis of a valuable product like carbon nano/microspheres from waste material, such as used cellulose acetate.

### Reaction Kinetics Model Results

The lumped kinetic model was utilized to calculate k_i_ values characterizing the hydrothermal conversion of CD at 210 °C. This model relies on mass concentrations of hydrochars and compounds, with the latter identified through NMR, all expressed in g/L. This approach does not require making assumptions about the molecular weight of hydrochar, which is necessary when dealing with models based on molar concentrations, as sometimes adopted in the literature concerning secondary char.[^49^, ^50^] The model was employed using various values of reaction order n≥1 for the formation of secondary char from HMF. This approach appears to offer greater precision in considering the formation of secondary char through HMF, involving repolymerization reactions characterized by multiple interactions and non‐elementary reactions.[^39^, ^51^]

**Figure 6:**
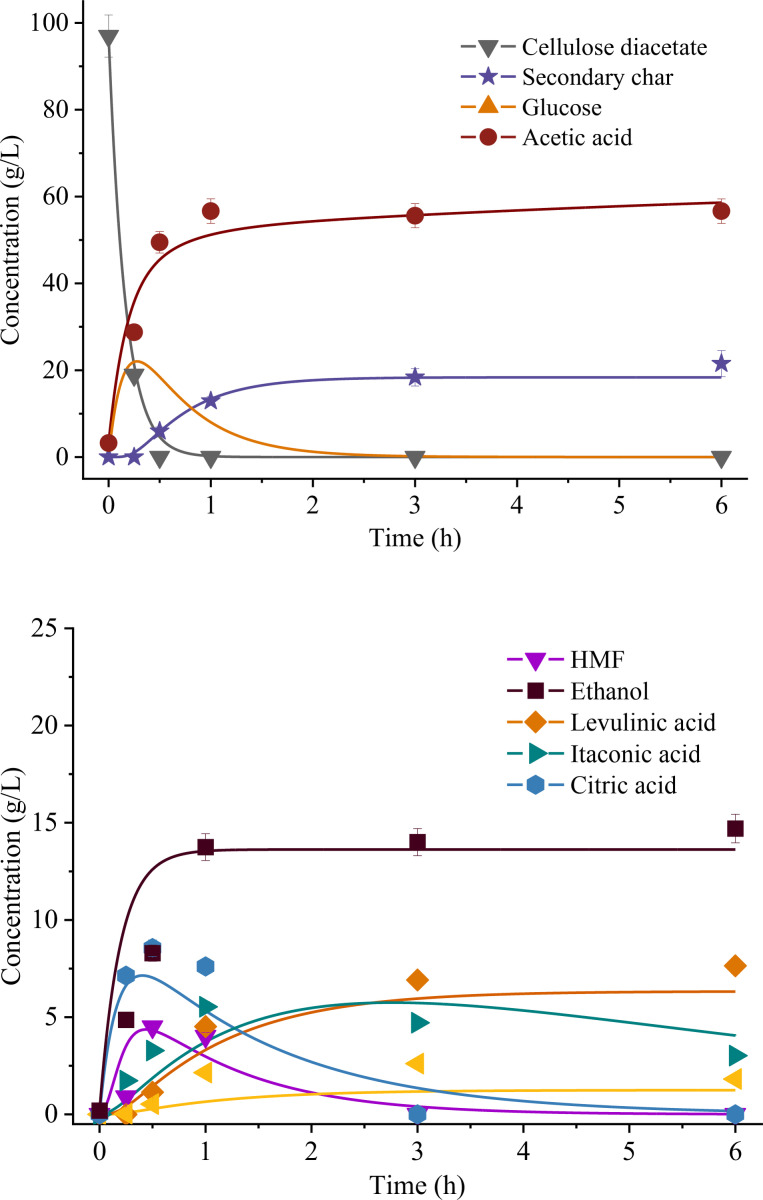
Comparison between model and experimental trends of compounds derived from the HTC of CD at 210 °C, considering n=2 for the reaction HMF → secondary char.

The predicted trends well align with the experimental data, as demonstrated in Figure 6. In comparison to the experimental results, the hydrochar was partitioned between residual CD, denoting unreacted CD decreasing its mass in the initial stage as evidenced by the SY minimum occurring at t=30 min (Figure 2b), and secondary char (≥30 minutes). This categorization aids in preventing overlap between these distinct solid phases, each characterized by markedly different kinetics–decomposition and repolymerization, respectively. This hypothesis is based on the experimental results above, which show that the cellulose acetate does not undergo carbonization during the first stage (before the minimum, Table 2) or, equivalently, does not undergo primary char formation mechanism. This discrepancy is evident in the modeled concentration trends (Figure 6a), showing residual CD approaching zero after 30 minutes, in contrast with the gradual increase of secondary char from zero to its peak concentration.

Table 3 shows the effect of n on k_1–10_, revealing that its variation slightly affects the results. Results show that reactions involving the decomposition of CD into glucose (k_1_), of glucose to HMF (k_2_), and CD to acetic acid (k_7_) have larger rates. Hence, these reactions proceed faster in the overall conversion process, in line with the higher predominance and ease of decomposition of CD and glucose in the system.

**Table 3:** Rate constants (k_i_) of the conversion of CD at 210 °C described by the lumped model in Figure 1, at different orders of reaction (n) for the reaction HMF → secondary char. The error function E equals 350.1 (n=1), 351.4 (n=1.5), and 353.4 (n=2).

Reaction rates (s^−1^)^[a]^	n=1	n=1.5	n=2
k_1_	6.6 ⋅ 10^−4^	6.5 ⋅ 10^−4^	6.5 ⋅ 10^−4^
k_2_	3.7 ⋅ 10^−4^	3.7 ⋅ 10^−4^	3.7 ⋅ 10^−4^
k_3_	1.7 ⋅ 10^−4^	1.6 ⋅ 10^−4^	1.6 ⋅ 10^−4^
k_4_	1.1 ⋅ 10^−3^	5.7 ⋅ 10^−4^	3.0 ⋅ 10^−4^
k_5_	7.1 ⋅ 10^−5^	6.2 ⋅ 10^−5^	5.5 ⋅ 10^−5^
k_6_	3.4 ⋅ 10^−4^	3.0 ⋅ 10^−4^	2.8 ⋅ 10^−4^
k_7_	6.7 ⋅ 10^−4^	6.7 ⋅ 10^−4^	6.8 ⋅ 10^−4^
k_8_	1.6 ⋅ 10^−4^	1.6 ⋅ 10^−4^	1.6 ⋅ 10^−4^
k_9_	1.9 ⋅ 10^−4^	1.9 ⋅ 10^−4^	1.9 ⋅ 10^−4^
k_10_	2.0 ⋅ 10^−4^	2.0 ⋅ 10^−4^	2.0 ⋅ 10^−4^

^[a]^ Except for k_4_, whose units are s^−1^ (g/L)^1−n^.

Despite the higher precision, using a n>1 complicates the ODE system, making it non‐linear and adding numerical challenges. Indeed, k_4_ (HMF to secondary char) decreases in response to a n increase, while all other k remain almost the same, indicating a good robustness of the model as well as the little effect of n on the entire system. To enhance precision while considering n, conducting further experimental studies involving various substrate concentrations would be beneficial. Additionally, the orders of magnitude for k align well with those reported in prior works employing alternative resolution techniques.[^49^, ^52^, ^53^]

This model could serve in effectively optimizing targeted compounds (like chemicals or secondary char) during the hydrothermal conversion within a biorefinery context. It could also provide a foundational framework for developing more intricate computational or machine learning models.[^54^, ^55^] In general, if appropriately calibrated, the model holds the potential for characterizing the conversion processes of other substrates, such as various biomass sources or cellulose acetates used in other sectors.

### Reaction Pathway and Potential Industrial Scenarios

**Figure 7:**
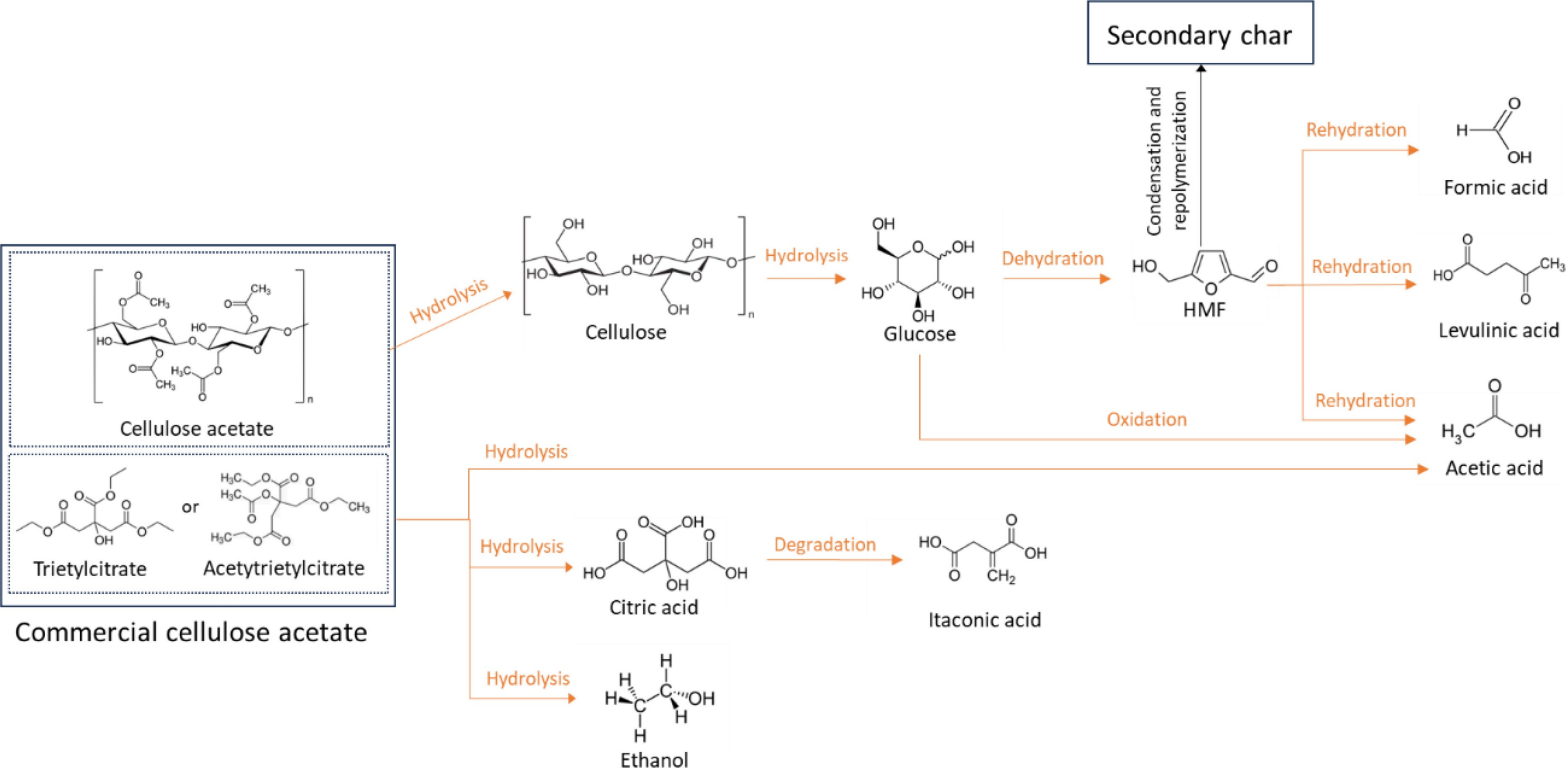
Simplified reaction scheme of the hydrothermal conversion of commercial cellulose acetate. Gas production is not reported.

In light of the experimental investigation, the main mechanisms and a simplified set of reaction pathways are outlined and schematically presented in Figure 7. During HTC, cellulose acetates undergo a complete re‐arrangement, leading to various products. The conversion starts with the release of additives from the material to the liquid phase at an onset condition depending on the specific additive and the form (shape, dimension) of the starting material. The formation of acetic acid (from acetyl groups of cellulose acetate or the degradation of the additive) leads to an acidic environment of pH 2.2–2.4 that enhances the cleavage of acetyl groups from the cellulose backbone as well as the hydrolytic cleavage of the cellulosic structure. Other acids (citric acid mainly) similarly contribute to the acidic nature of the environment. Thus, once the additive is released from the material matrix, two distinct pathways involving cellulose acetate and the additives can be identified as follows.

#### Regarding the *Cellulose Acetate Path*

1. Hydrolysis of cellulose acetate. The material undergoes an initial degradation process likely consisting of the simultaneous hydrolysis of acetyl groups (CH_3_CO−) linked to cellulose and beta‐glycosidic bonds constituting the cellulosic structure. Thus, the process is accompanied by a progressive material loss, resulting in a minimum point where less than 2.4 % of the starting material remains undissolved. At the same time, acetic acid and glucose monomers appear in the liquid phase. The release of acetic acid contributes to self‐catalyzing the reactions by decreasing the pH of the reaction environment;
2. HTC of glucose. Once produced, glucose monomers proceed through their typical HTC path. Thus, glucose partially isomerizes to fructose, and both become precursors for forming HMF and acetic acid.[^26^, ^39^, ^56^] HMF, as a pivotal intermediate, undergoes rehydration, leading to formic acid and levulinic acid, and simultaneously experiences partial repolymerization and condensation, forming secondary char in the form of carbon microspheres.

#### Regarding the *Additive Path*

1. Hydrolysis. Triethyl citrate breaks down into citric acid and ethanol, while triethyl acetyl citrate initially forms acetylated citric acid, hydrolyzing into citric acid, acetic acid, and ethanol;
2. Citric acid degrades into itaconic acid, likely through the loss of a carboxylic group and dehydration.

Together with the effectiveness in degradation of cellulose acetate, highly valuable products can be recovered, giving life to some biorefinery scenarios. Parameters could be adjusted to maximize the production of some specific compounds of interest. Among the most valuable and produced in relevant yields, we can identify:

- Secondary char in the form of microspheres, which form under harsher HTC conditions (>200 °C and >60 min). It is typically observed in sugar‐rich biomasses and can be a precursor of advanced carbons for a variety of applications, such as energy storage, catalysis, and water remediation.^[32]^
- HMF, a potential platform chemical,^[57]^ shows its maximum yield at intermediate HTC conditions (190–200 °C and 30–60 min).
- Itaconic acid and citric acid as high‐value chemicals for a potential recovery.[^41^, ^58^] They show complementary trends: itaconic acid forms under more severe conditions (>200 °C and >60 min), while citric acid at milder temperatures and shorter times.

In industrial scenarios, HTC could address the end‐of‐life of cellulose acetate residues, not only from the eyewear sector but also from other sources (cigarette butts being the most notable). If the substrate is dry, the necessary water could derive from a recirculation of the liquor itself, from other process streams or from nearby ′wet’ industries, such as food processing or organic waste companies. In this framework, the application of HTC is particularly viable if integrated within a biorefinery. The co‐HTC with other organic residues may also have synergistic effects,[^17^, ^59^] but may diminish the concentration of the chemicals of interest in the liquid phase as well, complicating their separation.

For cases in which recovering valuable liquid or solid products from the HTC of cellulose acetates is instead unviable, introducing HTC before anaerobic digestion could represent a valuable possibility. In this case, HTC would become a pre‐treatment before the conversion to biogas^[30]^: the high presence of sugar derivatives and acetic acid favors microorganisms’ metabolism, increasing the biogas yield and production rate.^[60]^ This scenario could allow the disposal of residual bioplastics with organic wastes and would avoid issues related to the relatively slow biodegradability of the cellulose acetates, especially if they are found in rather coarse forms. The acetyl groups, which HTC removes even at mild operating conditions, is indeed the main obstacle to the biodegradation of cellulose acetates.^[30]^ Another possibility is employing the liquor from this process (and possibly others) as a feedstock for the chemical^[61]^ or microbial^[62]^ synthesis of other bioplastics. However, the scientific works present in the literature in all these areas are still rather limited. The development of all these industrial scenarios requires intense R&D work to evaluate their scalability, merits, limitations, economic and environmental sustainability.

## Conclusions

Bioplastics have the sparkling potential to concretize circular economy principles and answer plastic related issues. Despite the novelty, they are not exempt from environmental challenges, like an established end‐of‐life pathway. This work proposes HTC as an option for disposing of bioplastic residues and recovering valuable products. In particular, it focuses on a specific class of bioplastics: cellulose acetates, whose specialized sector facilitates separate collection and disposal routes.

Results show that even a mild HTC (190 °C) effectively degrades the material, which undergoes an almost entire dissolution into the liquid phase, forming various chemicals. Then, as severity increases, a hydrochar rich in carbon and entirely consisting of microspheres (the so‐called secondary char) forms, while compounds like citric acid and itaconic acid form from the additives present in the original material. A comparison with pure compounds highlights the importance of these additives in the entire reaction environment: their derivatives help in material degradation by creating an acidic environment, catalyzing reactions, and structurally disrupting the material. From a biorefinery perspective, chemicals like itaconic acid and citric acid could be recovered together with secondary char to substantiate the circularity of the process. Finally, we also developed a kinetic model encompassing all the species detected by solid and liquid analyses. This model holds potential for facilitating further optimization endeavors and giving some insights behind the reaction mechanisms.

These findings suggest that HTC could be a valid option to enhance the end‐of‐life of residual cellulose acetates, fostering their circularity by converting them into added‐value products. The process would be especially justified within a biorefinery framework, with its actual configuration likely depending on various circumstances, such as the origin and composition of the available cellulose acetate(s) and the availability of liquid process streams to employ. Assessing the best configuration for the fruitful valorization of these residues also needs an accurate life cycle assessment, which is left for future scale‐up studies. Further research is also needed to ascertain the influence of different plasticizers, the effect of recirculating the process liquid to the HTC reactor, and the possibility of treating cellulose acetates with other residual bioplastics. The hydrothermal treatment of bioplastics is indeed a task thus far overlooked, but which could prove vital to ensure their virtuous disposal.

## Conflict of Interests

The authors declare no conflict of interest.

1

## Supporting information

As a service to our authors and readers, this journal provides supporting information supplied by the authors. Such materials are peer reviewed and may be re‐organized for online delivery, but are not copy‐edited or typeset. Technical support issues arising from supporting information (other than missing files) should be addressed to the authors.

Supporting Information

## Data Availability

The data that support the findings of this study are available from the corresponding author upon reasonable request.
