# Supplementary material for: Cellulose Acetates in Hydrothermal Carbonization: A Green Pathway to Valorize Residual Bioplastics
Source: ChemSusChem. 2024 Oct 18;18(2):e202401163. doi: 10.1002/cssc.202401163 (PMC11739857; doi:10.1002/cssc.202401163)
Supplement: Supplementary file 1 — Supporting Information [file CSSC-18-e202401163-s001.pdf]

ChemSusChem

Supporting Information

Cellulose Acetates in Hydrothermal Carbonization: A Green Pathway to Valorize Residual Bioplastics

Giulia Ischia, Filippo Marchelli,* Nicola Bazzanella, Riccardo Ceccato, Marco Calvi, Graziano Guella, Claudio Gioia,* and Luca Fiori

Cellulose acetates in hydrothermal carbonization: a green pathway to valorize residual bioplastics

Supplemental Information (for online publication only)

Table S1. Effect of time on the HTC, at 210 °C, of commercial cellulose diacetate (CD): mass yields and ultimate analyses on the deriving hydrochars.

**Time (h)**	**Mass yield (%)**			**Ultimate analyses (wt.%)**			
	**Solid**	**Gas**	**Liquid**	**C**	**H**	**N**	**O***
0	77.6	0.3	22.2	48.2	6.1	0.0	45.7
0.25	15.1	0.5	84.4	50.1	7.0	0.1	42.8
1	10.3	4.1	85.6	66.0	5.5	0.1	28.4
3	14.7	5.4	79.9	64.3	5.1	0.1	30.5
6	17.2	6.9	75.9	66.6	5.6	0.1	27.7

Table S2. Percentage differences between values calculated from NMR data and those measured via liquid yield (LY) and TOC analyzer. Data refer to the HTC of commercial cellulose diacetate (CD) and monoacetate (CM), at 1 h.

Temp (°C)	CD		CM	
	NMR vs. LY	NMR vs. TOC	NMR vs. LY	NMR vs. TOC
180	-52.1	-49.4	-20.9	-26.8
190	-5.0	-16.1	4.8	-12.9
200	2.7	-7.7	23.2	14.0
210	-7.1	-8.3	-	-
220	-6.2	8.5	-7.4	-8.6
250	-12.1	-9.3	-3.1	-1.1

Table S3. TOC data of the liquid phase recovered from HTC of commercial CD, at 210 °C.

Time (h)	0	1/4	1/2	1	3	6
TOC (g/L)	5.3	24.5	30.5	41.9	35.4	28.7

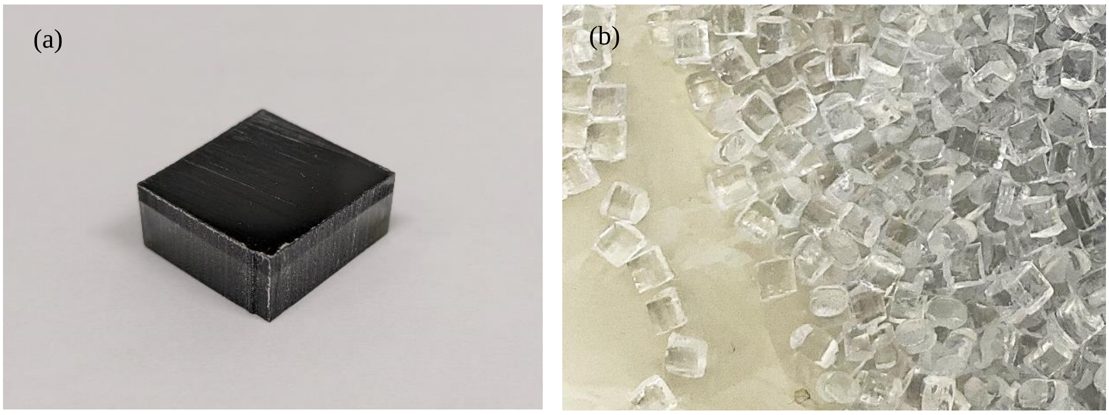

Fig. S1. Photos of the commercial cellulose acetates used for testing: (a) cellulose diacetate (CD) and (b) cellulose monoacetate (CM).

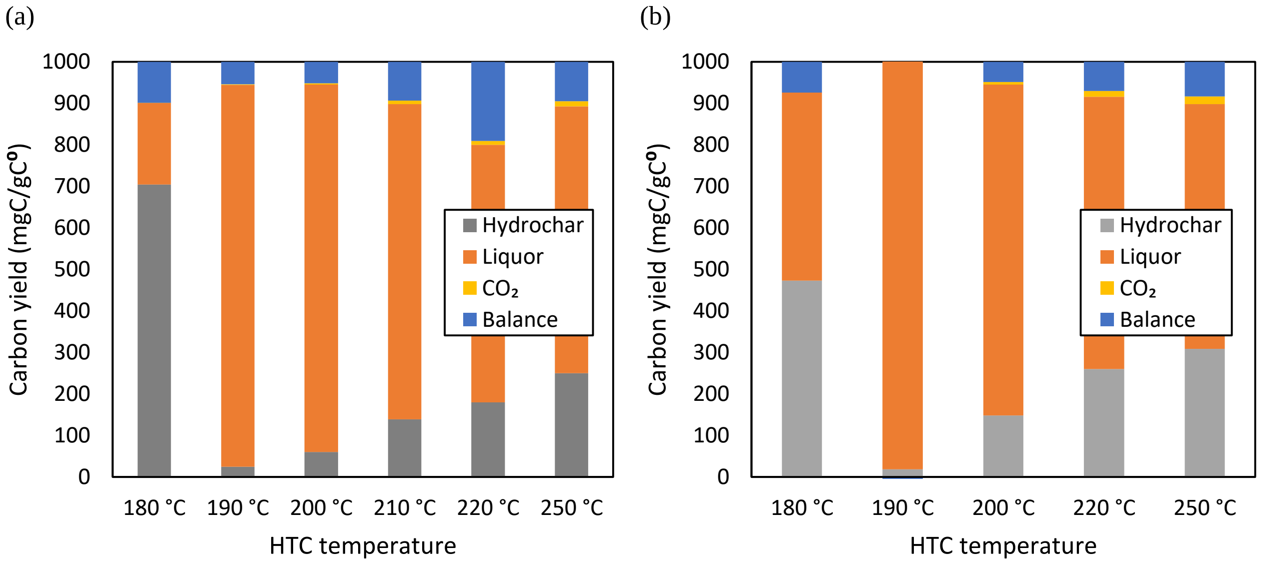

Fig. S2. Carbon yield for the tests on commercial cellulose acetates (a: diacetate; b: monoacetate). Data expressed in mg of carbon in the product per gram of carbon in the initial bioplastics. Average values are reported.

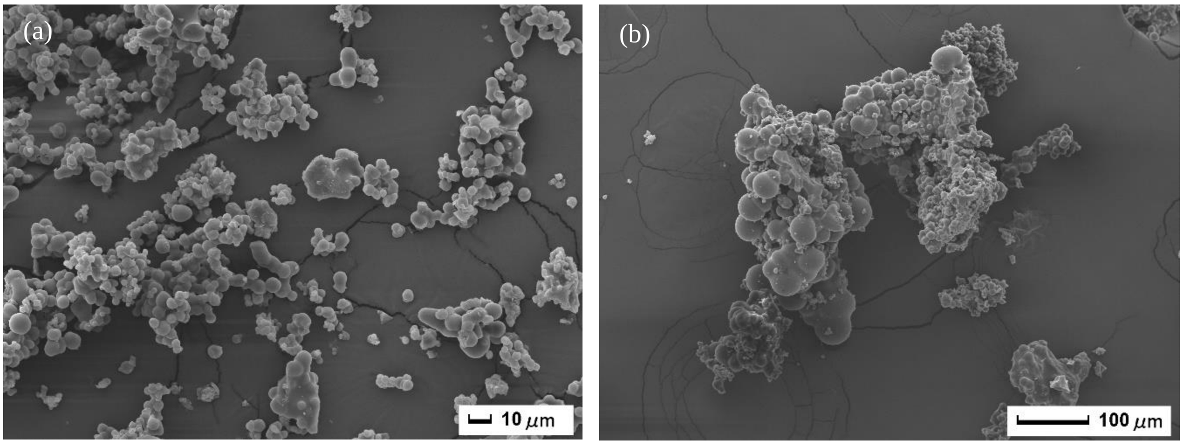

Fig. S3. SEM images of hydrochars produced at 250 °C 1 h from CM (a) and CD (b).

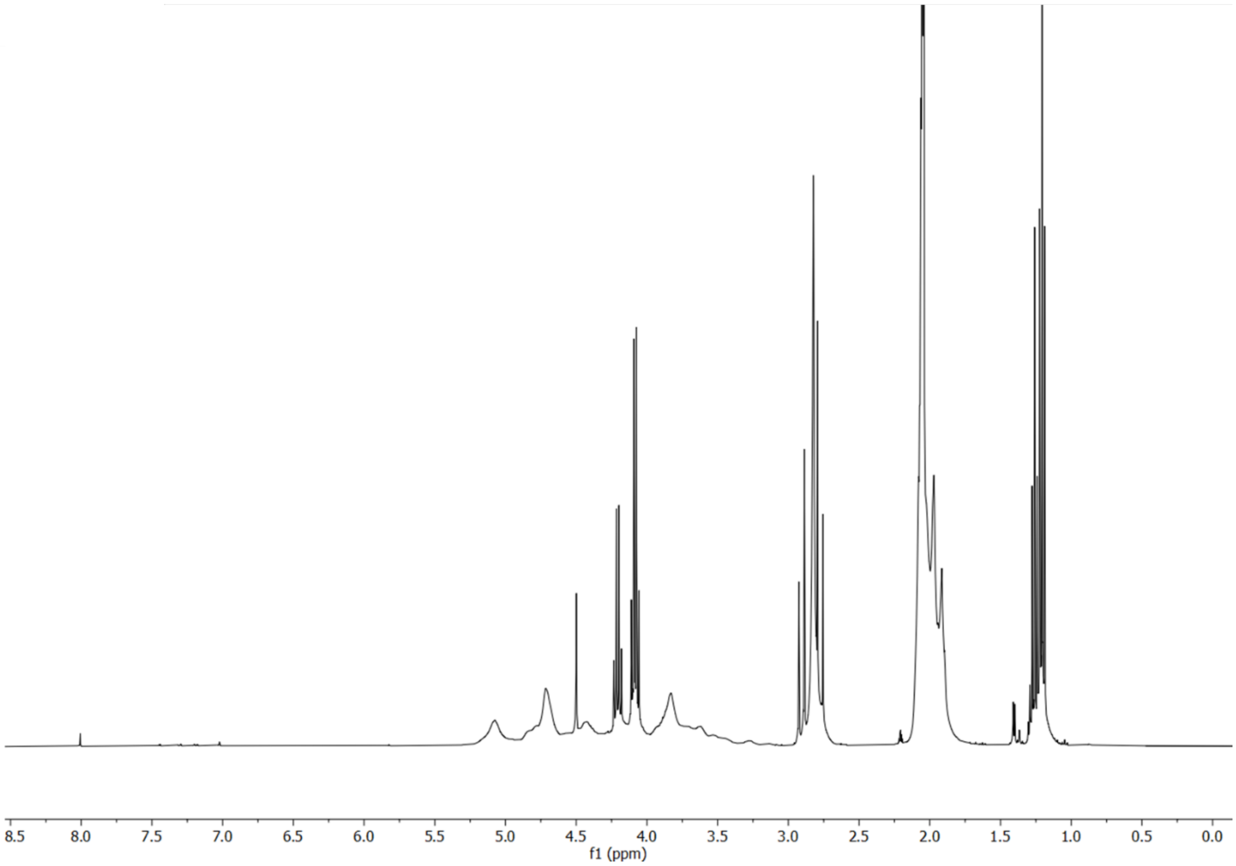

Fig. S4. ^{1}H-NMR of starting commercial CM in acetone-d_6.

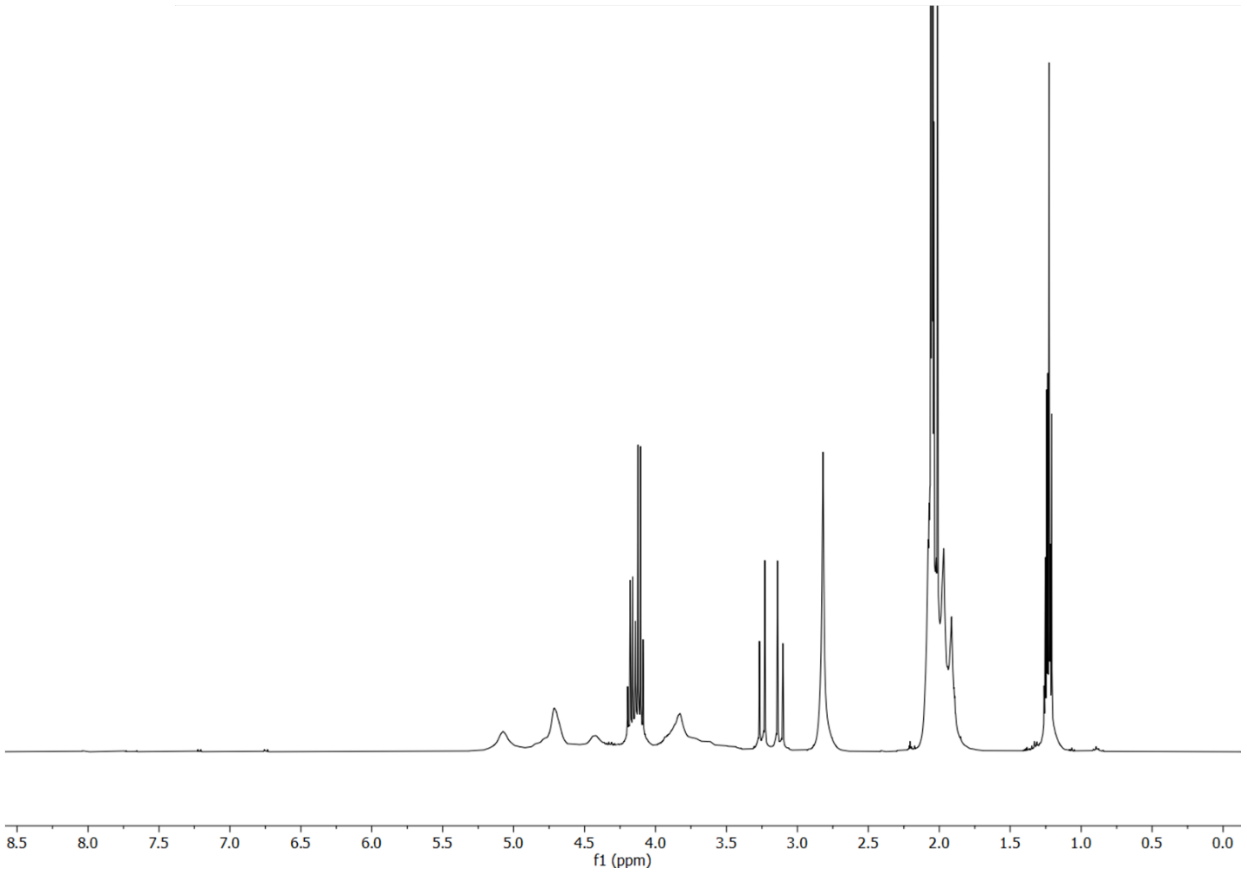

Fig. S5. ^{1}H-NMR of starting commercial CD in acetone-d_6.

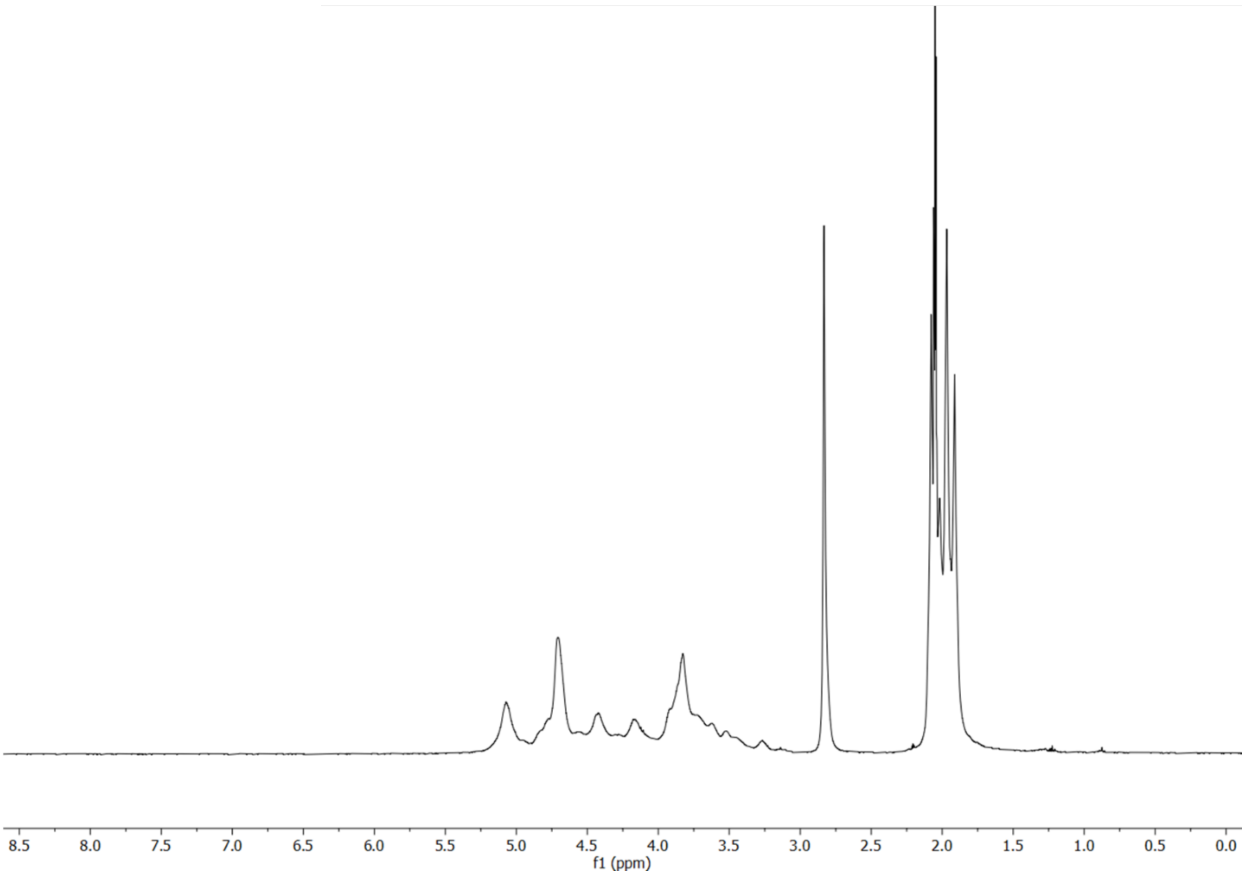

Fig. S6. ^{1}H-NMR of starting CD-pure in acetone-d_6.

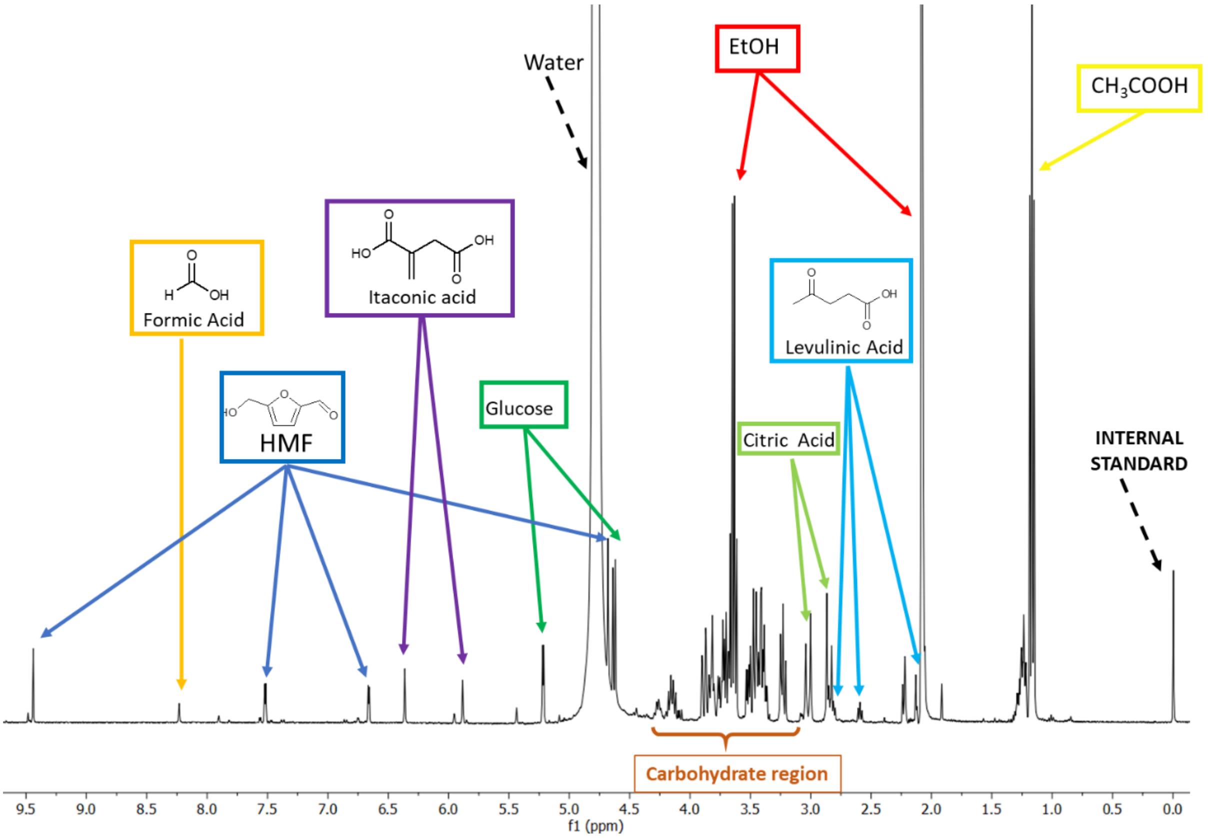

Fig. S7. Assignment by ^{1}H-NMR (D_2O) of the main components of the liquid fraction from HTC of CD at 200 °C for 1 hour.

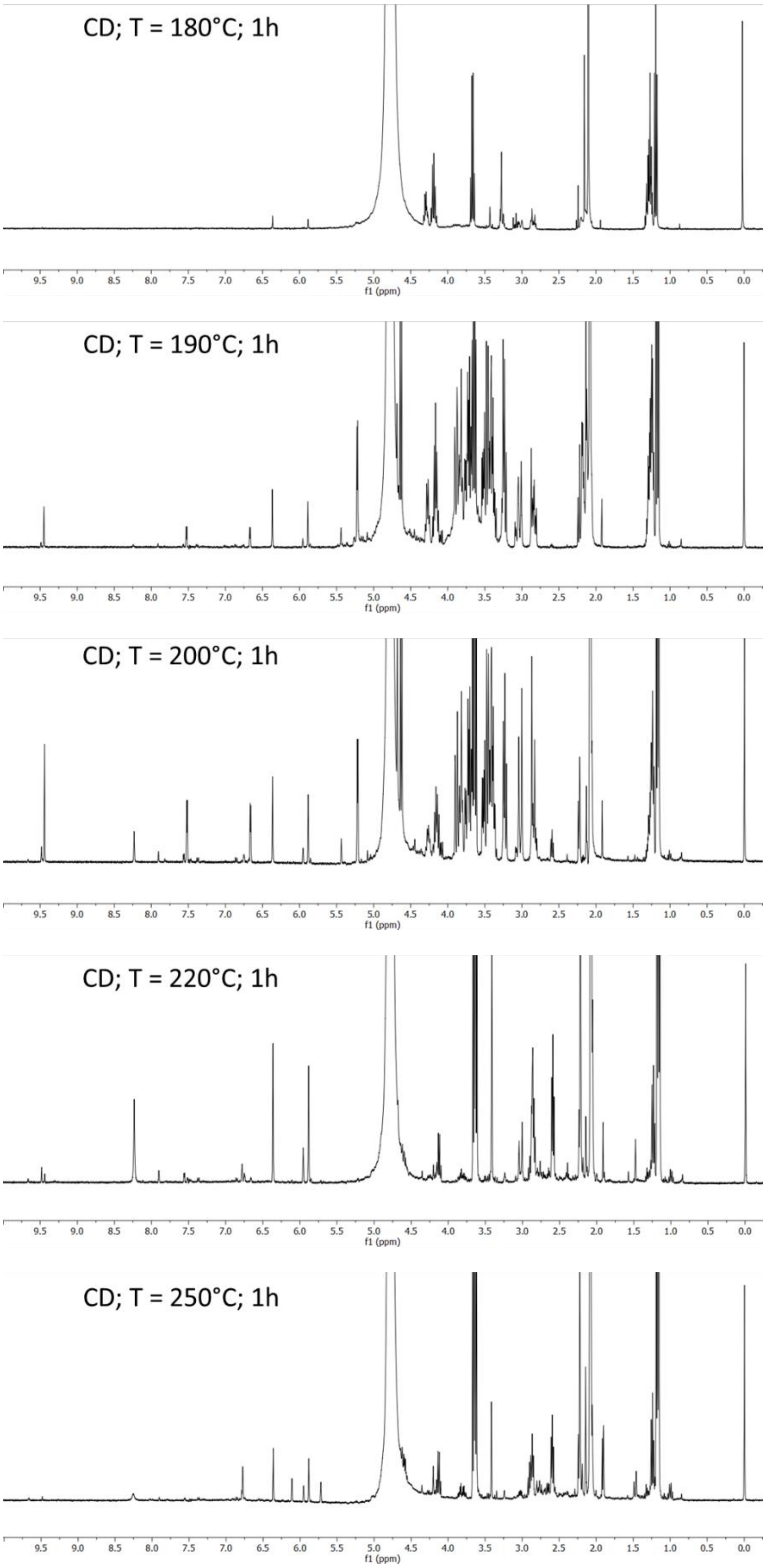

Fig. S8. ^{1}H-NMR (D_2O) of the liquid fraction from HTC of CD at different temperatures.

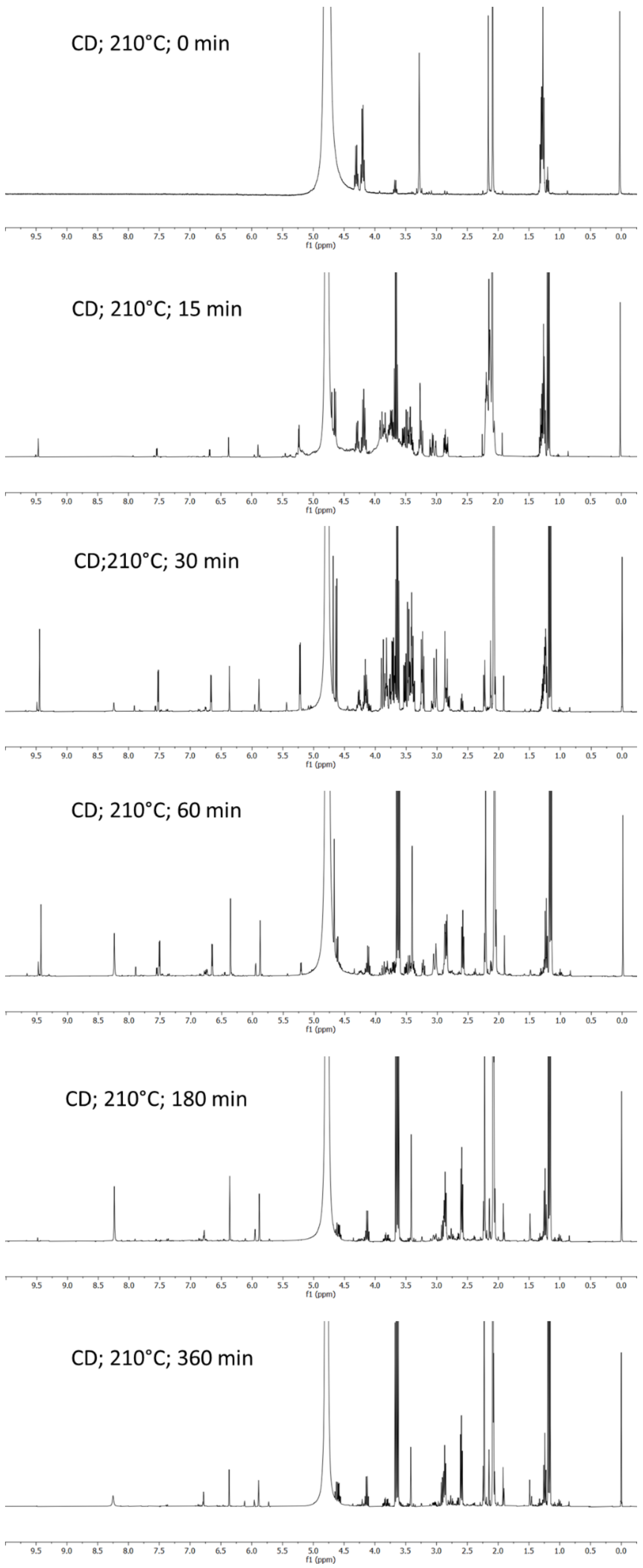

Fig. S9. ^{1}H-NMR (D_2O) of the time evolution of the liquid fraction from HTC of CD at 210 °C.
